# New Neuroimaging Findings in Enoyl-CoA Hydratase Short-Chain 1 (ECHS1) Deficiency

**DOI:** 10.7759/cureus.102392

**Published:** 2026-01-27

**Authors:** Hiroko Tada, Keiko Ichimoto, Kei Murayama, Tomohide Goto, Jun-ichi Takanashi

**Affiliations:** 1 Department of Pediatrics, Chibaken Saiseikai Narashino Hospital, Narashino, JPN; 2 Department of Metabolism, Chiba Children's Hospital, Chiba, JPN; 3 Department of Diagnostics and Therapeutics of Intractable Diseases, Intractable Disease Research Center, Graduate School of Medicine, Juntendo University, Tokyo, JPN; 4 Department of Neurology, Kanagawa Children's Medical Center, Yokohama, JPN; 5 Department of Pediatrics, Tokyo Women's Medical University Yachiyo Medical Center, Yachiyo, JPN

**Keywords:** arterial spin labeling, cerebellum, echs1, leigh syndrome, magnetic resonance spectroscopy, valine

## Abstract

Enoyl-CoA hydratase short-chain 1 (ECHS1) variants are among the most common causes of Leigh syndrome. A five-year-old boy with ECHS1 deficiency initially presented with acute encephalopathy during the neonatal period. The patient had a high serum lactate level and a normal lactate/pyruvate ratio. Diffusion-weighted imaging showed reduced diffusion in the peri-rolandic subcortical white matter on day 3 and in the entire cortex and subcortical white matter on day 7. The patient subsequently presented with poor feeding, hypotonia, nystagmus, cerebellar ataxia, hearing loss, and strabismus. At one year of age, neuroimaging revealed reduced diffusion, hyperperfusion on arterial spin labeling, and increased lactate on magnetic resonance (MR) spectroscopy in the cerebellum. Cerebellar lesions have not previously been reported as imaging findings of ECHS1 deficiency except in one previous report of a patient with the same ECHS1 variant. Therefore, the ECHS1variant may specifically involve the cerebellum.

## Introduction

The enoyl-CoA hydratase short-chain 1 (ECHS1) gene is crucial for the metabolism of the essential amino acid valine. Defects in this gene cause the accumulation of toxic substances and disturbances in energy production; they can also cause Leigh syndrome (MIM #256000) [1]. Clinically, patients with ECHS1 deficiency exhibit variable combinations of developmental delay, hypotonia, ataxia, and episodes of acute encephalopathy. Neuroimaging typically reveals lesions in the basal ganglia and brainstem; however, cerebellar involvement is considered extremely uncommon [2-4].

Herein, we report a case of ECHS1 deficiency in a patient whose neuroimaging findings changed over time and included cerebellar lesions at approximately one year of age.

## Case presentation

**Figure 1:**
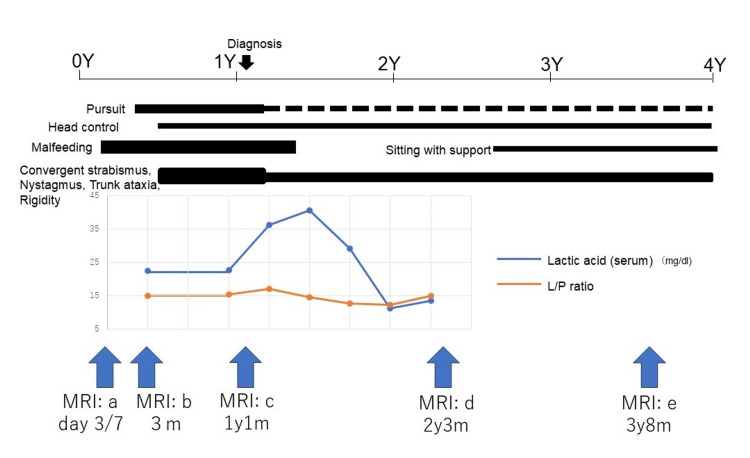
The longitudinal course of the clinical symptoms, changes in serum lactate and the L/P ratio, and timing of magnetic resonance studies Lactate levels were highest at one year and one month (40 mg/dL), and the L/P ratios were all within normal limits (<20). MRI: magnetic resonance imaging; L/P ratio: lactate/pyruvate ratio

The patient is now a five-year-old Japanese boy, who was born to healthy parents. He was born at 39 weeks of gestation via normal delivery and weighed 2,546 g. On day 3 after birth, the patient was admitted with acute encephalopathy due to persistent convulsions and impaired consciousness. Blood tests performed on admission showed slightly elevated serum lactate (27.9 mg/dL; reference range: 4.0-19.2) and pyruvate (2.02 mg/dL; reference range: 0.3-0.9) levels with a normal lactate/pyruvate (L/P) ratio of 13.8 (reference range: 10-20). No abnormalities were detected in the newborn screening tests. The automatic auditory brainstem response test suggested left-sided hearing loss. The patient was intubated during days 3-7 after birth, improved slowly, and was discharged on day 21 (Figure 1).

**Figure 2:**
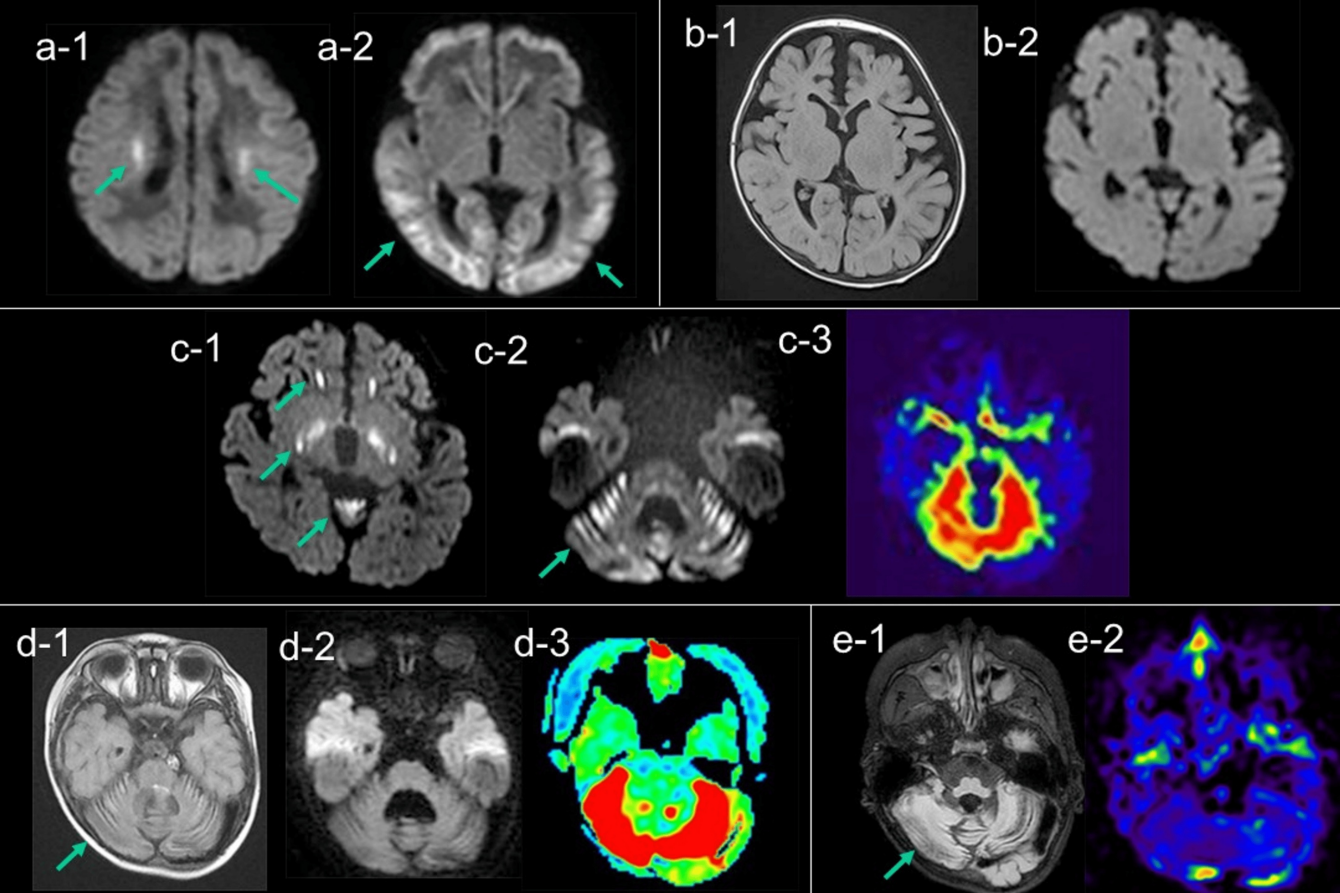
Temporal changes in MRI (a) DWI showed reduced diffusion in the peri-rolandic subcortical white matter (a-1: arrow) on day 3, followed by that in the subcortical white matter on day 7 (a-2: arrow). (b) FLAIR (b-1) and DWI (b-2) at three months revealed mild cerebral atrophy without obvious parenchymal signal abnormalities. (c) At one year and one month, DWI showed reduced diffusion in the globus pallidus, substantia nigra, anterior periventricular white matter, and cerebellar cortex (c-1 and c-2: arrow). ASL showed hyperperfusion in the cerebellum (c-3). (d) At two years and two months, FLAIR showed high signal intensity in the atrophic cerebellar cortex (d-1: arrow). DWI showed no diffusion abnormalities (d-2); however, ASL still showed hyperperfusion in the cerebellum (d-3). (e) At three years and eight months, FLAIR showed progressive cerebellar atrophy (e-1: arrow) with hypoperfusion on ASL (e-2). MRI: magnetic resonance imaging; DWI: diffusion-weighted imaging; FLAIR: fluid-attenuated inversion recovery; ASL: arterial spin labeling

Diffusion-weighted imaging (DWI) on day 3 revealed hyperintensity in the peri-rolandic subcortical white matter (Figure 2, a-1), which spread to the entire cortex, subcortical white matter, and corpus callosum on day 7 (Figure 2, a-2). The lesions disappeared on day 21. Despite extensive serum and cerebrospinal fluid (CSF) workup, the underlying cause of the acute encephalopathy remained uncertain. He was able to control his head at three months of age and roll over at six months. He slowly exhibited ataxia of the trunk, rigidity of the limbs, nystagmus, and strabismus (Figure 1). Magnetic resonance imaging (MRI) at three months revealed mild cerebral atrophy without obvious parenchymal signal abnormalities (Figure 2, b-1 and b-2).

**Figure 3:**
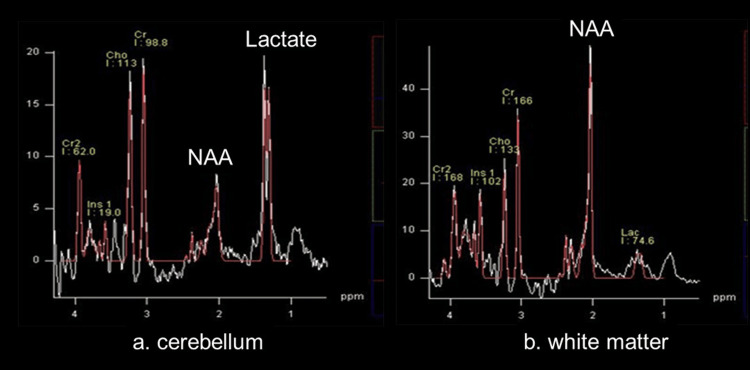
Magnetic resonance spectroscopy at one year and one month In the cerebellum (a), a marked decrease in NAA levels was observed with a prominent lactate peak. In the white matter (b), magnetic resonance spectroscopy showed reduced NAA without obvious lactate. NAA: N-acetylaspartate

Laboratory test results at 11 months showed high lactate and pyruvate concentrations in the blood and CSF, with a normal L/P ratio, and elevated 2-methyl-2,3-dihydroxybutyric acid levels in the urine (Figure 1). This led to a suspected diagnosis of ECHS1 deficiency. The diagnosis was confirmed by whole-exome analysis, which revealed a compound heterozygous variant of ECHS1 (c.176 A>G/c.23 T>C) at one year and three months of age. T2 hyperintensity and high signal intensity on DWI were observed in the globus pallidus, substantia nigra, and white matter around the anterior horn of the lateral ventricle at one year and one month of age (Figure 2, c-1). Additionally, T2-weighted imaging (T2WI) and DWI revealed a high signal intensity lesion in the cerebellum (Figure 2, c-2), with hyperperfusion on arterial spin labeling (ASL) (3D-ASL; TR/TE=8000/11 ms; post-labeling delay (PLD)=1500 ms) (Figure 2, c-3) and markedly elevated lactate levels on magnetic resonance (MR) spectroscopy (TR/TE 5000/30 msec, quantitatively analyzed using LCModel) (Figure 3).

After making a definite diagnosis of ECHS1 deficiency at one year and three months of age, a low-protein and valine-restricted diet (plasma valine concentrations under 85 µmol/L) and vitamin cocktail therapy (biotin, vitamins B, C, and E, and coenzyme Q10) were initiated under the guidance of a metabolic specialist. The patient's clinical condition and lactate and pyruvate levels were monitored every one to three months.

After initiating dietary and vitamin therapy, no further neurological deterioration was observed. Motor function stabilized, and the patient achieved new developmental milestones, such as sitting and standing with support, at five years of age (Figure 1).

At two years and two months, MRI showed cerebellar atrophy and high signal intensity in the cerebellar cortex on fluid-attenuated inversion recovery (Figure 2, d-1) with normal signal on DWI (Figure 2, d-2); however, ASL still showed cerebellar hyperperfusion (Figure 2, d-3). At three years and eight months, MRI revealed progressive cerebellar atrophy with ASL hypoperfusion (Figure 2, e-1 and e-2).

## Discussion

The most important aspects of this case were the previously unreported DWI findings during the neonatal period and cerebellar lesions at one year of age.

The patient presented with hyperlactatemia with a normal L/P ratio during the neonatal period, wherein DWI showed reduced diffusion that spread over time from the peri-rolandic subcortical white matter (day 3) to the entire cortical and subcortical lesions (day 7). The mechanism by which ECHS1 deficiency causes hyperlactatemia with a normal L/P ratio is thought to be because methacryl-CoA and acryl-CoA accumulate due to ECHS1 deficiency, resulting in the dysfunction of the electron transport chain and pyruvate dehydrogenase complex (PDHC) activity, the latter of which consequently leads to hyperlactatemia with a normal L/P ratio, similar to that observed with PDHC deficiency [5]. Neuroimaging of patients with ECHS1 deficiency during the neonatal period [6-9] revealed cortical dysplasia, periventricular pseudocysts, hypoplasia of the corpus callosum, and abnormalities of the basal ganglia, in addition to diffuse white matter abnormalities. These imaging findings are similar to those observed in neonatal PDHC deficiency [10], suggesting that neonatal ECHS1 deficiency, similar to neonatal PDHC deficiency, develops in utero. In contrast, in our case, no evidence of brain dysplasia was observed; the migration of lesions with reduced diffusion suggested a postnatal onset. In neonatal patients with hyperlactatemia, with a normal L/P ratio and reduced diffusion in the subcortical white matter, ECHS1 should be considered; this might facilitate early diagnosis and treatment [8].

Masnada et al. [7] reported 19 cases of ECHS1 deficiency, of which seven cases developed from late infancy until two years, named the slowly progressive infantile form, and showed MRI lesions in the periventricular white matter and basal ganglia [9,10]. The MRI findings at the age of one year and one month in this case were consistent with those of the slowly progressive infantile form showing white matter and basal ganglia lesions. However, cerebellar lesions have never been reported except for in this case and another one with the same ECHS1 variants (c.176A>G/c.23T>C) [11]. MR spectroscopy at one year and one month of age showed a marked lactate peak in the cerebellum, which was not observed in the white matter or basal ganglia, suggesting that mitochondrial dysfunction in the cerebellum was particularly severe. It is reasonable to assume that the hyperperfusion in the cerebellum observed on ASL might have compensated for energy depletion resulting from mitochondrial dysfunction. The reason why ECHS1 with the variants caused a more vulnerable impact on cerebellar neurons than basal ganglia or white matter remains unclear. Further studies are needed to confirm whether cerebellar lesions are specific to this mutation.

## Conclusions

We report a case of ECHS1 deficiency in a boy whose neuroimaging findings changed over time. This case presents new neuroimaging findings of ECHS1 deficiency, including early cortical-subcortical diffusion restriction and subsequent cerebellar involvement. These findings might serve as diagnostic clues; however, they should be interpreted with caution until further confirmed in additional cases.

## References

[1] Peters H, Buck N, Wanders R (2014). ECHS1 mutations in Leigh disease: a new inborn error of metabolism affecting valine metabolism. Brain.

[2] Sato-Shirai I, Ogawa E, Arisaka A (2021). Valine-restricted diet for patients with ECHS1 deficiency: divergent clinical outcomes in two Japanese siblings. Brain Dev.

[3] Ganetzky R, Stojinski C (2019). Mitochondrial short-chain enoyl-CoA hydratase 1 deficiency. GeneReviews® [Internet].

[4] Murofushi Y, Ochiai K, Yasukochi M (2024). Increased ketone levels as a key magnetic resonance spectroscopic findings during acute exacerbation in ECHS1-related Leigh syndrome. Radiol Case Rep.

[5] Ogawa E (2020). ECHS1 disorder and HIBCH disorder as abnormalities of valine metabolism. Mitochondria and Diseases, Genetic Medicine 35.

[6] Ganetzky RD, Bloom K, Ahrens-Nicklas R, Edmondson A, Deardorff MA, Bennett MJ, Ficicioglu C (2016). ECHS1 deficiency as a cause of severe neonatal lactic acidosis. JIMD Rep.

[7] Masnada S, Parazzini C, Bini P (2020). Phenotypic spectrum of short-chain enoyl-Coa hydratase-1 (ECHS1) deficiency. Eur J Paediatr Neurol.

[8] Muntean C, Tripon F, Bogliș A, Bănescu C (2022). Pathogenic biallelic mutations in ECHS1 in a case with short-chain enoyl-CoA hydratase (SCEH) deficiency-case report and literature review. Int J Environ Res Public Health.

[9] Bedoyan JK, Yang SP, Ferdinandusse S (2017). Lethal neonatal case and review of primary short-chain enoyl-CoA hydratase (SCEH) deficiency associated with secondary lymphocyte pyruvate dehydrogenase complex (PDC) deficiency. Mol Genet Metab.

[10] Van der Knaap MS (2005). Magnetic Resonance of Myelination and Myelin Disorders. Chapter 28; Leigh syndrome and mitochondrial leukoencephalopathies.

[11] Uchino S, Iida A, Sato A, Ishikawa K, Mimaki M, Nishino I, Goto YI (2019). A novel compound heterozygous variant of ECHS1 identified in a Japanese patient with Leigh syndrome. Hum Genome Var.

